# Effects of dental anxiety and anesthesia on vital signs during tooth extraction

**DOI:** 10.1186/s12903-024-04404-5

**Published:** 2024-05-29

**Authors:** Diya Zhang, Shenglai Li, Rui Zhang

**Affiliations:** 1 Dental Department, Sir Run Run Shaw Hospital, Zhejiang University School of Medicine, 3 East Qingchun Road, Hangzhou, 310016 Zhejiang China; 2 Department of Oral and Maxillofacial Surgery, Stomatology Hospital, Zhejiang University School of Medicine, Hangzhou, 310016 China; 3 Dongfang College, Zhejiang University of Finance and Economics, Hangzhou, China

**Keywords:** Dental anxiety, Tooth extraction, General anesthesia, Local anesthesia, Dental anxiety scale

## Abstract

**Background:**

Anxiety is common preceding tooth extraction; hence, it is crucial to identify patients with dental anxiety (DA) and to manage DA. This study assessed the level of DA and influencing factors in tooth extraction patients in a dental hospital in China and changes in their blood pressure (BP) and heart rate (HR) during the tooth-extraction procedure.

**Methods:**

The study was a cohort study. The Dental Anxiety Scale (DAS) was used to assess the level of DA of 120 patients before tooth extraction. A Demographics and Oral Health Self-Assessment Form was used to assess factors influencing DA. The correlations of DAS scores with HR and BP were measured. The effects of local anesthesia and general anesthesia on HR and BP were also compared using a Datex-Ohmeda anesthesia monitor to detect HR and BP continuously before and after anesthesia. Independent sample t-tests, OLS multiple regression model and one-way analysis of variance were applied to analysis the results.

**Results:**

Based on the DAS score, 12.5% of the participants were identified as suffering from DA. DA was related to age, gender, and the self-assessment of oral health. The DAS score was correlated with increased BP (*P* < 0.05). BP showed an overall upward trend after local anesthesia, while it was generally stable after general anesthesia. The systolic BP at 4 and 5 min and the HR at 2 and 4 min increased remarkably (*P* < 0.05) after local anesthesia compared with those before anesthesia. The HR and BP of patients under local anesthesia were generally higher than those of patients under general anesthesia were during the operation.

**Conclusions:**

The prevalence of DA in adults was 12.5% in this study population. DA was related to gender, age, and the self-assessment of oral health. The score of DAS was correlated with BP. Compare to local anesthesia, general anesthesia can make the vital signs of tooth extraction patients more stable.

**Supplementary Information:**

The online version contains supplementary material available at 10.1186/s12903-024-04404-5.

## Introduction

Dental anxiety (DA), also known as dental fear, refers to all psychological and physiological variations of a more or less strong but not pathological feeling of fear in conjunction with a dentist’s appointment or stimuli relating to dental treatment [1–3]. It has been reported that DA is one of the most common barriers to dental treatment, and that approximately 6-25% of the population suffer severe DA, which is associated with dental non-attendance [3–6]. Persons who avoid dental treatment have more oral diseases and have greater adverse effects on their daily lives from poor dental health, such as their quality of life, appearance, and self-esteem [7, 8]. Research has found that trying to manage patients with DA is a source of fairly substantial stress for many dentists [9]. Moreover, people who delay dental visits for a prolonged time (even when they experience considerable pain) are more likely to have extensive problems that require more complex and complicated treatment [10]. Therefore, DA is an emerging clinical problem that needs to be solved with the development of dentistry.

Tooth extraction is a very basic and widely used operation in dental surgery, and it is also a common treatment for some dental diseases. However, it can cause local tissue damage, bleeding, swelling, pain and other reactions, and can lead to fluctuations in heart rate, blood pressure, and body temperature [11, 12]. Tooth extraction, as an invasive procedure in dental practice, is known to be among the top five most frightening dental procedures [13]. Patients are more worried and anxious about tooth removal than they are about pain or helplessness. Losing a tooth is also a notable stimulus that provokes anxiety, and DA is usually higher among patients having tooth extractions [13, 14]. Despite improvements in dental care, patients usually still have a fear of tooth extraction. This is especially true for children and patients with systemic diseases who have poor pain tolerance, cardiac function, blood vessel elasticity, and other adverse factors. Research aimed at understanding the anxiety of patients and how to ensure their safety before tooth extraction is a relatively new topic.

It is obvious that the anxiety and fear of patients in the dental office should be controlled as much as possible. In order to do this, identifying anxious individuals, affecting factors and their appropriate management becomes of vital importance in clinical practice. One commonly measure is the Corah’s Dental Anxiety Scale (DAS), which is a 4-item tool that asks patients to estimate their level of anxiety in four different dental situations [15]. The questionnaires used in the present study included the DAS to measure the level of DA, and questions about demographic and oral health history to assess the relationship between the DAS score and various factors that influence it. Heart rate (HR) and blood pressure (BP) were recorded before and after tooth extraction. As anesthesia is essential in tooth extraction, the effects of local anesthesia (LA) and general anesthesia (GA) on the HR and BP of patients undergoing tooth extraction were also compared and analyzed.

This study aimed firstly, to assess the level of DA and influencing factors in tooth extraction patients; secondly, to evaluate the changes in their blood pressure (BP) and heart rate (HR) during the tooth-extraction procedure; and finally, to compare the effect of local anesthesia and general anesthesia on these parameters. The hypothesis of this study is that many people suffer from DA; DA varies with certain demographic characteristics and oral health; the DAS score has correlation with vital signs and general anesthesia can make the vital signs of DA patients more stable.

## Materials and methods

### Study design and participants

We designed a cohort study to investigate the prevalence of DA and the correlation between DAS score and vital signs including BP and HR during tooth extraction. The participants were recruited from patients who underwent tooth extraction between August 2023 and November 2023 at the Department of Oral and Maxillofacial Surgery of the Affiliated Stomatology Hospital, School of Medicine, Zhejiang University (Hangzhou, China). According to the formula for determining the sample size of the survey: n = Z^2^σ^2^/d^2^ (n: Represents the required sample size; Z: The Z-statistic at the 95% confidence level is 1.96; σ: The overall standard deviation is generally taken as 0.5, d: Sampling error). When d = 5-10%, *n* = 96–384. A total of 120 outpatients undergoing tooth extraction were randomly selected, including 58 males and 62 females. The inclusion criteria were: (1) patients 18 to 60 years-old, and (2) no relevant systemic diseases (American Society of Anesthesiologists’ classifications ASA I and ASA II). The exclusion criteria consist of pregnant women and patients with malignant diseases or mental disabilities. All of the participants were informed consent and asked to finish two questionnaires. The BP and HR were recorded before and after the tooth extraction. The study protocol was approved by the ethics committees of Stomatology Hospital, Zhejiang University School of Medicine.

### Questionnaire measures

#### Dental anxiety scale (DAS) [16, 17]

The DAS consists of 4 questions to measure the degree of DA in 4 situations: preparing for a dental visit, waiting in the dentist’s office for treatment, sitting in the dental chair for drilling, and getting ready in the dental chair for scaling. The total score ranges from 4 (not anxious) to 20 (extremely anxious). Patients with a DAS score ≥ 13 were considered to have DA (Appendix 1).

#### Demographics and oral Health Self-Assessment Form (DOF) [17]

In addition to the DAS, the outpatients were asked to complete a form, which obtained information about demographics and oral health. The demographic information included gender (Male/Female), age (18–30/31–60), level of education (High school or below/ College or above). Two measures of oral health were obtained: a self-assessment of oral health (Healthy/Unhealthy), and a self-assessment of dental treatment needs (Required/Not required) (Appendix 2).

### BP and HR measures

The outpatients rested for 5 min after lying on the dental chair and their BP and HR were recorded using a digital BP meter by calculating the mean of many times measure. The LA was 2% lidocaine with 1:100,000 ratio of adrenaline for all participants. The administration of LA and the tooth extraction were conducted by one single dental professional at the Department of Oral and Maxillofacial Surgery of the Affiliated Stomatology Hospital, School of Medicine, Zhejiang University (Hangzhou, China).

### Procedures

**Fig. 1:**
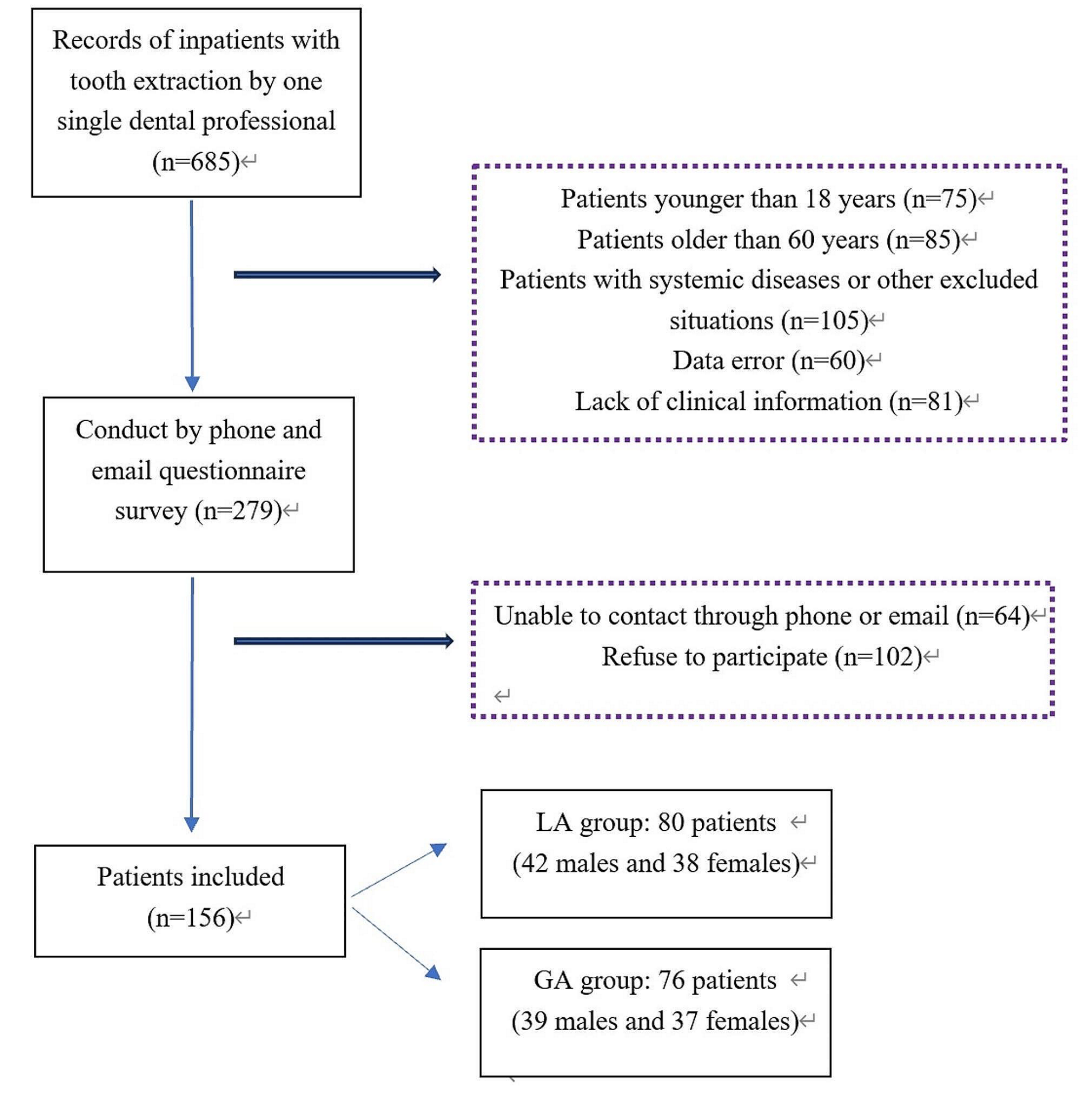
Flowchart of the screening process. LA: local anesthesia; GA: and general anesthesia

We designed a retrospective study to determine the effect of anesthesia on vital signs including BP and HR during tooth extraction (Fig. 1). The information of 685 inpatients with tooth extraction by one single dental professional from January 2023 to August 2023 at the Department of Oral and Maxillofacial Surgery was collected, and a total of 279 patients were included based on the inclusion and exclusion criteria. An informed consent was obtained by all the participants by phone and/or email. At last, 156 (55.91%) inpatients were admitted including 80 patients (42 males and 38 females) who received LA, and 76 patients (39 males and 37 females) who received GA (Fig. 1). The HR and BP records of the participants during tooth extraction were collected from medical record database. Inpatients who received LA were given a local nerve injection of 2% lidocaine hydrochloride containing a ratio of 1:100,000 adrenaline. Inpatients who received GA were given an intravenous anesthetic, intubated through the oral trachea, and local infiltration anesthesia with a 2% lidocaine hydrochloride injection containing a ratio of 1:100,000 adrenaline at the gingival flap in the mouth after the GA took effect. An anesthesia monitor (Datex-Ohmeda; Crosswell Co. Ltd, Tokyo, Japan) was used to obtain real-time BP and HR data during the tooth extraction procedure.

### Statistical analyses

Descriptive and non-parametric bivariate statistics were used to describe the sample, and continuous variables with normalized distribution were presented as mean with standard deviation. Independent sample t-tests were performed to compare the mean total DAS scores between the groups, regression analysis was performed using an OLS multiple regression model, with DAS score as the dependent variable, and one-way analysis of variance (ANOVA) was applied to evaluate the different effects of LA and GA on vital signs including HR and BP. All statistical analyses were conducted using SPSS version 19.0 (SPSS, Chicago, IL, USA) and *P* < 0.05 was set as the level of statistical significance for all the statistical analyses.

## Results

### Results of questionnaires

The average DAS score was (9.02 ± 3.11). The highest score was 18 and the lowest was 4, and 12.5% of the patients were considered to have DA. The relationships of the DAS score with gender, age, educational level, self-assessment of oral health, and self-assessment of dental treatment needs are shown in Table 1. Compared to males and older adults, females and younger adults were more likely to have DA. Self-assessment of oral health showed significant relationship with DA. The participants who perceived their teeth unhealthy were more anxious. While educational level and self-perceived dental treatment needs were not significantly associated with DA. All the variables mentioned above were included in an OLS multiple regression analysis (Table 2). Gender (95% CI: 0.400, 2.418), and self-assessment of oral health (95% CI: 0.949, 3.122) were positively correlated with DAS score. While age (95% CI: -3.081, -1.015) was negatively correlated with DAS score.

**Table 1:** Demographic and clinical features of patients and their mean DAS score

Variables	Categorization	Number	Score (mean ± SD)	*P*-value
Gender	Male	58	8.19 ± 2.77	0.004^*^
	Female	62	9.79 ± 3.24	
Age (years)	18**–**30	55	9.91 ± 2.55	0.003^*^
	31–60	65	8.26 ± 3.36	
Educational level	High school or below	40	9.50 ± 2.94	0.231
	College or above	80	8.78 ± 3.19	
Self-assessment of oral health	Healthy	75	8.25 ± 3.12	0.000^*^
	Unhealthy	45	10.29 ± 2.67	
Self-perceived dental treatment needs	Required	77	9.35 ± 2.99	0.116
	Not required	43	8.42 ± 3.28	

DAS: Dental Anxiety Scale, SD: standard deviations. Independent sample t-tests were performed to compare the mean total DAS scores between the groups. ^*^P < 0.05

**Table 2:** OLS multiple regression model for analyzing DAS

Variables	Coefficient	Std. Err.	*P*	[95% Conf.	Interval]
Gender	1.409	0.509	0.007^*^	0.400	2.418
Age	-2.048	0.521	0.000^*^	-3.081	-1.015
Educational level	-0.591	0.565	0.298	-1.711	0.529
Self-assessment of oral health	1.409	0.549	0.000^*^	0.949	3.122
Self-perceived dental treatment needs	-0.313	0.549	0.570	-1.401	0.775
_cons	8.648	2.167	0.000^*^	4.355	12.941

DAS: Dental Anxiety Scale, Std. Err: standard Error. Coefficient is an unstandardized coefficient. ^*^P < 0.05

### The correlation DAS score and vital signs

The relationships of DAS with increased HR and BP are shown in Table 3. The DAS score had a weak non-significant negative correlation with increased HR (*P* > 0.05), but a significant positive correlation with increased BP including SBP and DBP (*P* < 0.05).

**Table 3:** Correlation between increased HR and BP with DAS score (mean ± SD)

DAS Score	Increased HR	Increased SBP	Increased DBP
8.89 ± 2.68	13.41 ± 7.51	27.68 ± 14.15	19.92 ± 8.06
r	-0.03	0.24	0.03
*P*	0.67	0.01^*^	0.03^*^

HR = heart rate (Times / minute), SBP = systolic blood pressure (mmHg), DBP = diastolic blood pressure (mmHg), DAS = Dental Anxiety Scale, SD = standard deviations. Increased HR/BP = maximum value - minimum value. ^*^P < 0.05

### Changes in BP before and after anesthesia

**Fig. 2:**
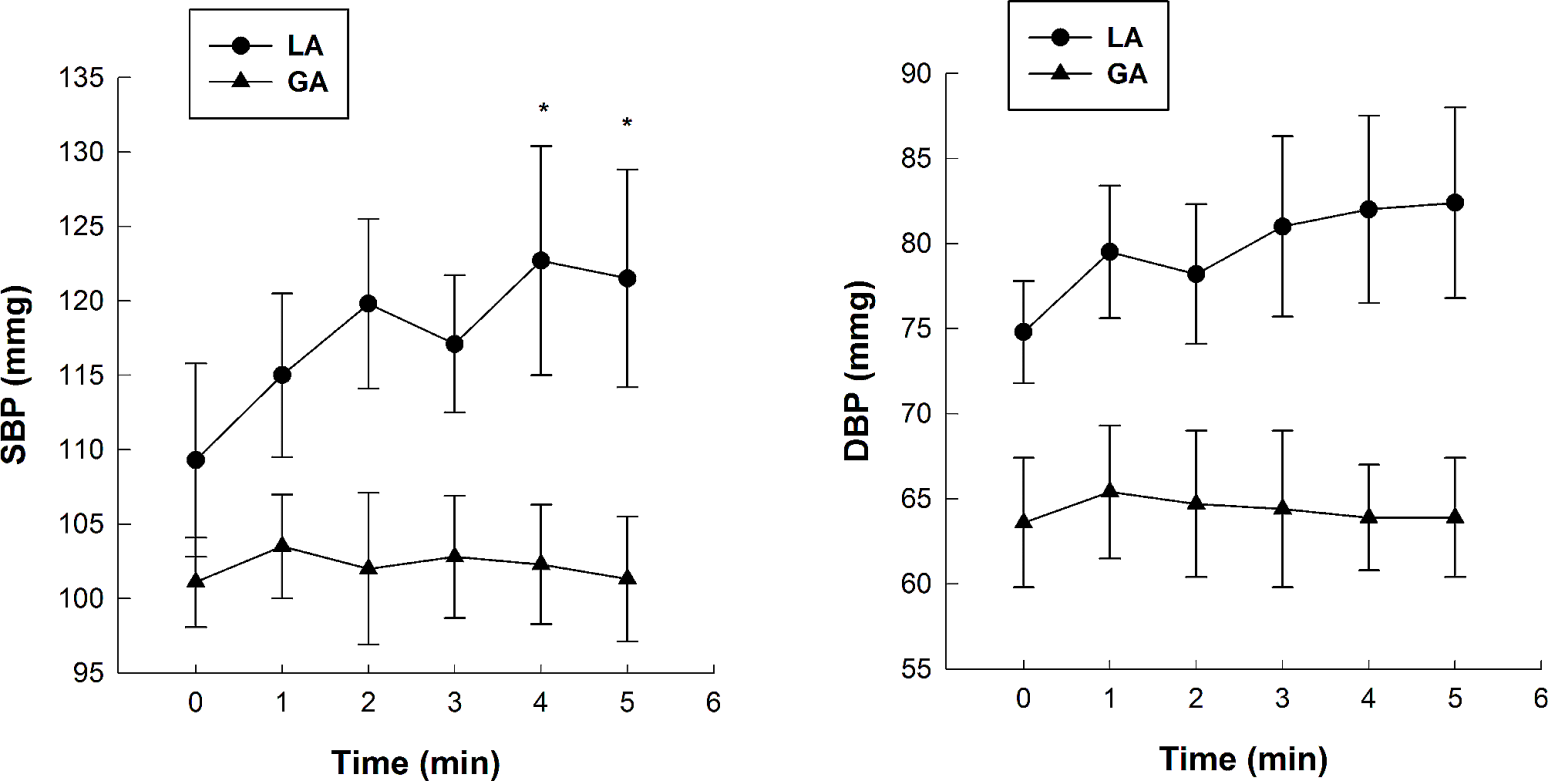
Changes in the blood pressure of tooth-extraction patients before and after anesthesia. Real-time SBP and DBP during the tooth extraction procedure was recorded by an anesthesia monitor. ^*^P < 0.05, LA group compared with GA group

Both SBP and DBP showed an overall upward trend after local anesthesia (LA) (Fig. 2). SBP remarkably increased (*P* < 0.05) at 4 and 5 min after LA compared with its level before anesthesia. There was no significant difference in DBP after LA compared with its level before anesthesia (*P* > 0.05). However, there was no significant difference (*P* > 0.05) in SBP or DBP, which were generally stable during the operation in the GA group. The SBP and DBP of patients under LA were generally higher than those under GA during the operation.

### Changes in HR before and after anesthesia

**Fig. 3:**
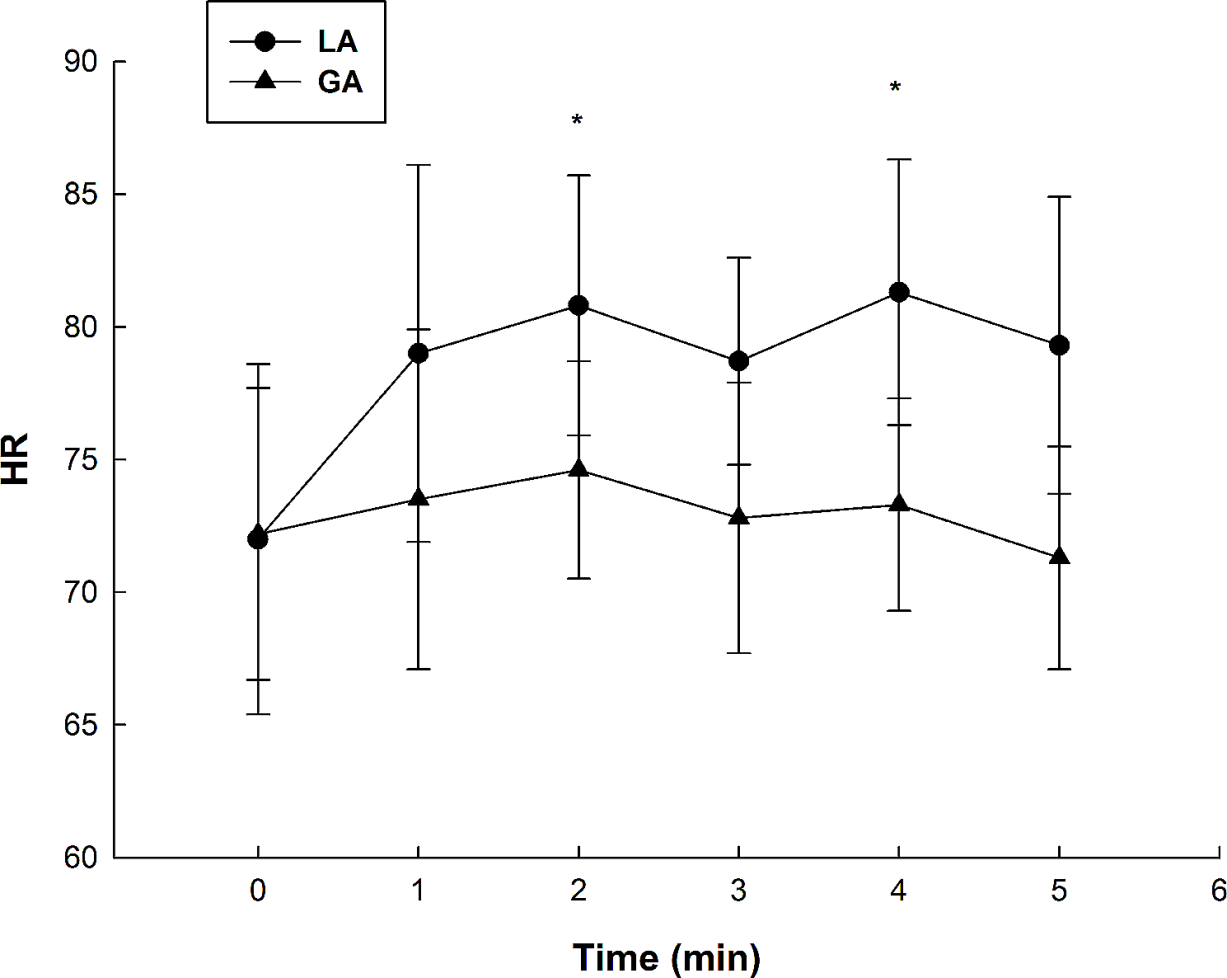
Changes in the heart rate of tooth-extraction patients before and after anesthesia. Real-time HR during the tooth extraction procedure was recorded by an anesthesia monitor. ^*^P < 0.05, LA group compared with GA group

Overall, HR was higher after LA than GA (Fig. 3). HR was obviously higher at 2 min and 4 min after LA than before LA (*P* < 0.05). Whereas, after GA, HR fluctuated a little, but there was no statistically significant difference in HR before and after anesthesia (*P* > 0.05).

## Discussion

The present study was carried out to assess the level of DA, influencing factors and effects of DA and anesthesia on vital signs during tooth extraction. The results shows that the DA level was 12.5% in this study population; DA was related to gender, age, and the self-assessment of oral health; the score of DAS was correlated with BP; and compare to local anesthesia, general anesthesia can make the vital signs of tooth extraction patients more stable.

Corah’s DAS is one of the most popular psychological scales used to diagnose DA. The DAS has been translated into multiple languages because of its reliability and cross-cultural validity [18, 19]. Its advantages are its brevity, simplicity, being easy to complete, and its cost-effectiveness for research [15, 20]. DA, described as an emotion that helps organisms defend against a variety of threats, arises from a dysregulation of the body’s normal defensive response [21]. This emotion leads to an increase in the secretion of endogenous catecholamines, which results in a rise in HR and BP during extraction. The hemodynamic changes caused by emotional stress mask the alterations caused by exogenously active catecholamines. The main brain area involved is the hypothalamic-pituitary-adrenal axis. Cortisol and catecholamines are released in response to various stimuli, such as: fear, anxiety, and the expectation or experience of pain [22, 23]. It is difficult to describe the prevalence of DA accurately due to study design, sampling methods, setting, and the administration of questionnaires, as well as cultural attitudes and socio-economic variations. Among adults, the prevalence of DA varies widely in the research literature, with its reported prevalence ranging from 4.2% to over 50% [3, 6, 24]. In the present study, the DA prevalence was 12.5%. The methodological diversity or geographical variation can result in this discrepancy to some extent.

Researches have shown that the prevalence of DA varies with certain demographic characteristics [25, 26]. In this study, gender, age, and level of education were selected to analysis the correlation of demographics with DA according to the former theory and the research literatures. Comparison between genders, the DAS scores of females were generally higher than those of males (Table 1). Gender was positively correlated with DAS score (Table 2). Our study results align with the majority of available studies [27–29]. The plausible explanation for gender difference could be a combination of emotional and social factors. Women usually had a greater readiness to acknowledge feelings or anxiety while men usually felt embarrassed and tend to hide their anxiety toward dental procedures [27]. Furthermore, it was observed that older people had lower DAS scores than younger people (Table 1). Age was negatively correlated with DAS score (Table 2). It is controversial about the correlation of age and DA. It was found that among age groups, patients who were 24–34 years-old show the highest DAS scores, and the incidence of DA was higher in young people than it was in old people, possibly because there was a general reduction in anxiety as people get older [30]. Yet, some studies, such as Erten et al., have found no statistically significant differences between the mean DAS scores of different age groups [31]. We also found that the individual’s judgment of their oral health was an important influencing factor of DAS scores and people with poor self-assessments of their oral health had higher DAS scores which was consistent with a study conducted in Germany [32]. Whereas DAS scores were not related to level of education or self-perceived dental treatment needs in the present study.

We then examined the changes in BP and HR of outpatients before and after tooth extraction, and the correlations of the increased BP and HR with DA. The results showed that DAS scores had a non-significant negative correlation with increased HR (*P* > 0.05). In contrast, DAS scores had a significant positive correlation with increased BP (*P* < 0.05) (Table 3). Furthermore, we used a Datex-Ohmeda anesthesia monitor for continuous measurement of the inpatient’s BP and HR, in order to compare and analyze the effects of LA and GA on the BP and HR of inpatients undergoing tooth extraction. From this experiment, it can be seen that vital signs including BP and HR changed after anesthesia. The tension and anxiety before anesthesia, as well as the pain stimulation during anesthesia, could all cause an increase in sympathetic nerve excitability, leading to the release of more catecholamines from the medulla of the renal gland, which activate receptors in the heart and blood vessels, result in HR and BP rise, and blood oxygen saturation decrease. If not corrected timely, it could cause arrhythmia, myocardial ischemia, and heart failure [33]. The results showed that the use of GA with patients undergoing tooth extraction had less impact on the vital signs than the use of LA. Therefore, the selection of GA for tooth extraction is relatively safer and more reliable than LA, especially for patients with DA. Though pain was eliminated in the patients given LA, the thought of pain may always be present. DA results in nerve excitement and increased catecholamines in the human body, which is manifested as tension, anxiety, and discomfort in tooth extraction. Even after receiving LA, patients have a rapid HR and increased BP [34, 35]. GA inhibits the central nervous system of the entire body, and it not only eliminates the feeling of pain during the operation, it also makes its memory temporarily disappear. Thus, patients under GA will not have psychological problems, such as fear and depression, during the tooth extraction. Therefore, for some patients, such as those with DA, GA is safer than LA, which makes is more suitable for DA patients.

The prevalence of DA, the factors associated with dental anxiety and the relationship with vital sign, which could be of significance to dental practitioners and dental health services. If they are aware about above situations among their patients, they can anticipate patient’s behavior and be better prepared to take measures to help alleviate anxiety, thereby enabling them to pursue better patient management strategies and treatment making. Thus, it is meaningful to further research these topics. Before extraction, we should take effective measures to ensure the surgery is conducted smoothly and safely for DA patients. First of all, the communication between dentists and patients is important. The language of psychological care can reduce the fears of DA patients and enable them to cooperate with the dentist. Secondly, it would be much better to use a Datex-Ohmeda anesthesia monitor or an electrocardiogram monitor during the tooth extraction of DA patients, which can not only detect changes in vital signs of patients at any time, but also improve the patient’s sense of self-security, which alleviates the anxiety of patients to some extent. Thirdly, we should choose suitable anesthetic drugs and methods of anesthesia to reduce the pain of patients, so as to reduce the occurrence of dental phobia and enable patients to receive early, comfortable, and high-quality treatment.

Limitations of the study are insufficient operability of research variables and self-assessment questionnaires can be biased when eliciting responses and fewer anxiety-related factor were assessed, and failure to consider the sampling process in the retrospective clinical study design. In the future studies, larger and more diverse samples should be evaluated, and more anxiety-related factors and Randomized Controlled Trial can be further investigated.

## Conclusion

This study found the prevalence of DA in adults was 12.5%. DA was related to gender, age and the self-assessment of oral health. The DAS score had a weak negative and non-significant correlation with increased HR, but it had a significant positive correlation with increased BP. Compare to local anesthesia, general anesthesia can make the vital signs of tooth extraction patients more stable. Since DA is a barrier for regular dental treatment, it is necessary for clinicians and dental health services to identify the patient with DA, select suitable method and well-equipment, which would improve people’s psychological and oral health.

### Electronic supplementary material

Below is the link to the electronic supplementary material.

Supplementary Material 1

Supplementary Material 2

Supplementary Material 3

## Data Availability

The datasets generated and/or analysed during the current study are not publicly available due to the clinical data and privacy of patients but are available from the corresponding author on reasonable request.
