# Supplementary material for: Effects of dental anxiety and anesthesia on vital signs during tooth extraction
Source: BMC Oral Health. 2024 May 29;24:632. doi: 10.1186/s12903-024-04404-5 (PMC11134746; doi:10.1186/s12903-024-04404-5)
Supplement: Supplementary file 1 — Supplementary Material 1 [file 12903_2024_4404_MOESM1_ESM.docx]

**Dental Anxiety Scale (DAS)**

Name______________________________________________ Date___________

1. If you had to go to the dentist tomorrow for a check-up, how would you feel about it?

a. I would look forward to it as a reasonably enjoyable experience.

b. I wouldn't care one way or the other.

c. I would be a little uneasy about it.

d. I would be afraid that it would be unpleasant and painful.

e. I would be very frightened of what the dentist would do.

2. When you are waiting in the dentist's office for your turn in the chair, how do you feel?

a. Relaxed.

b. A little uneasy.

c. Tense.

d. Anxious.

e. So anxious that I sometimes break out in a sweat or almost feel physically sick.

3. When you are in the dentist's chair waiting while the dentist gets the drill ready to begin

working on your teeth, how do you feel?

a. Relaxed.

b. A little uneasy.

c. Tense.

d. Anxious.

e. So anxious that I sometimes break out in a sweat or almost feel physically sick.

4. Imagine you are in the dentist's chair to have your teeth cleaned. While you are waiting

and the dentist or hygienist is getting out the instruments which will be used to scrape

your teeth around the gums, how do you feel?

a. Relaxed.

b. A little uneasy.

c. Tense.

d. Anxious.

e. So anxious that I sometimes break out in a sweat or almost feel physically sick.

Scoring the DAS (this information is not printed on the form that patients see)

a = 1, b = 2, c = 3, d = 4, e = 5 Total possible = 20

The total score ranges from 4 (not-anxious) to 20 (extremely anxious). Patients with a DAS score ≥ 13 were considered to have DA
