# Supplementary material for: Effects of dental anxiety and anesthesia on vital signs during tooth extraction
Source: BMC Oral Health. 2024 May 29;24:632. doi: 10.1186/s12903-024-04404-5 (PMC11134746; doi:10.1186/s12903-024-04404-5)
Supplement: Supplementary file 2 — Supplementary Material 2 [file 12903_2024_4404_MOESM2_ESM.docx]

**STROBE Statement**

**Title and abstract**

**1(a):** In the “**abstract**” “***Methods***” line 1

**1(b):** In the “**abstract**” “***Methods*** and ***Results***”

**Introduction**

**2 Background/rationale:** In the “**introduction**” paragraph 1 and paragraph 2

**3 Objectives:** In the “**introduction**” paragraph 3

**Methods**

**4 Study design:** In the “**Material and methods**” “***Study design and participants***” line 1-2 and “***The effects of anesthesia on HR and BP*”** line 1-2 and Fig. 1

**5 Setting:** In the “**Material and methods**” “***Study design and participants***” line 2-5; line 10-13 and “***The effects of anesthesia on HR and BP*”** line 2-5

**6 Participants (a):** In the “**Material and methods**” “***Study design and participants***” line 2-5 and “***The effects of anesthesia on HR and BP*”** line 2-5

**(b):** not applicable

**7 Variables:** Gender, Age, Educational level, Self-assessment of oral health, Self-perceived dental treatment needs. In the “**Material and methods**” **“*Questionnaire measures*”** and Table 1.

**8 Data sources/ measurement:** In the **“Material and methods”**

**9 Bias:** The subjects were recruited according to the inclusion criteria and exclusion criteria (in the “**Material and methods**” “***Study design and participants***” line 10-13); and the operations of dental extraction were conducted by one surgeon (in the “**Material and methods**” “***BP and HR measures***” line 4)

**10 Study size:** In the “**Material and methods**” “***Study design and participants***” line 6-10 and in the “**Material and methods**” “***The effects of anesthesia on HR and BP***” line 2-8 and Fig. 1

**11** **Quantitative variables:** In this study quantitative variables including: DAS Score, SBP, DBP, HR. The DAS score ranges from 4 (not anxious) to 20 (extremely anxious). Patients with a DAS score ≥ 13 were considered to have DA (In the “**Material and methods**” “***Questionnaire measures***” “Dental Anxiety Scale (DAS)” line 3-5); BP and HR of outpatients were recorded using a digital BP meter by calculating the mean of many times measure (In the “**Material and methods**” “***BP and HR measures***” line 1-2); An anesthesia monitor (Datex-Ohmeda) was used to obtain real-time BP and HR data during the tooth extraction procedure and the average values of inpatients were calculated at every time point under local or general anesthesia. DAS Score, SBP, DBP, HR were all presented as mean with standard deviation (In the “**Material and methods**” “***Statistical analyses***” line 2).

**12 Statistical methods (a):** In the “**Material and methods**” “***Statistical analyses***” line 3-9. We used independent sample t-tests to compare the mean total DAS scores between the groups. With DAS score as the dependent variable, an OLS multiple regression model was used to perform regression analysis. One-way analysis of variance (ANOVA) was applied to evaluate the different effects of LA and GA on HR and BP. All statistical analyses were conducted using SPSS version 19.0 (SPSS, Chicago, IL, USA) and *P* < 0.05 was set as the level of statistical significance for all the statistical analyses.

**(b)** **(c) (d)** **(e):** not applicable

**Results**

**13 Participants (a):** The specific screening process is shown in **Fig. 1.**

**(b):** The reasons for non-participation at each stage were shown in **Fig. 1.**

**(c):** as suggested, we use a flow diagram (Fig. 1).

**14 Descriptive data (a):** In the “**Results**” “***Results of questionnaires***” line 1-6 and Table 1

**(b) (c):** not applicable

**15 Outcome data:** In the “**Results**” “***Results of questionnaires***” line 1-2. The average DAS score was (9.02±3.11). The highest score was 18 and the lowest was 4, and 12.5% of the patients were considered to have DA.

**16 Main results:**

**(a):** 95% confidence interval was showed in Table 2

**(b):** Patients with a DAS score ≥ 13 were considered to have DA

**(c):** not applicable

**17 Other analyses:** In the “**Results**” “***Changes in BP before and after anesthesia***” and “***Changes in HR before and after anesthesia***”

**Discussion**

**18 Key results:** In the “**Discussion**” paragraph 1, paragraph 2 and paragraph 3.

**19 Limitations:** In the “**Discussion**” paragraph 4

**20** **Interpretation:** In “**Discussion**” paragraph 1, line 11-17, and paragraph 2, line 6-20

**21 generalisability:** In the “**Conclusion**”

**22 Funding:** none
