# Supplementary material for: Effects of dental anxiety and anesthesia on vital signs during tooth extraction
Source: BMC Oral Health. 2024 May 29;24:632. doi: 10.1186/s12903-024-04404-5 (PMC11134746; doi:10.1186/s12903-024-04404-5)
Supplement: Supplementary file 3 — Supplementary Material 3 [file 12903_2024_4404_MOESM3_ESM.docx]

**Oral Health Self-Assessment Form**

| Variable | Categorization |
| --- | --- |
| Gender | Male |
|  | Female |
| Age (years) | 18**-**30 |
|  | 31-60 |
| Educational level | High school or below |
|  | College or above |
| Self-assessment of oral health | Healthy |
|  | Unhealthy |
| Self-perceived dental treatment  needs | Required |
|  | Not required |
